# Social housing improves dairy calves' performance in a competition test

**DOI:** 10.3168/jdsc.2023-0378

**Published:** 2023-08-12

**Authors:** Malina Suchon, Thomas Ede, Bianca Vandresen, Marina A.G. von Keyserlingk

**Affiliations:** 1 Animal Welfare Program, Faculty of Land and Food Systems, University of British Columbia, Vancouver, BC, Canada V6T 1Z4; 2 Swine Teaching and Research Center, University of Pennsylvania School of Veterinary Medicine, Kennett Square, PA 19348

## Abstract

On most dairy farms, calves are housed individually until weaning. However, depriving calves of an early social environment impairs behavioral development. We studied the effect of early-life social housing on calves' competitive skills. In this study, Holstein heifers were pseudorandomly assigned to either individual housing (n = 9) or pair housing (with a nonfocal companion, n = 9) at the age of 11 d. After 14 d of housing treatment, calves underwent a competition test for milk access against a group-reared calf; consisting of 2 test sessions per day for 5 d (session duration: 74.42 ± 2.29 s; mean ± standard error). Pair-housed calves performed better than individually housed calves: throughout the competition days, individually housed calves increased their latency to approach the milk bottle and decreased their time spent drinking in contrast to pair-housed calves, which exhibited stable latencies to reach the milk bottle and increased their time drinking. To control for the influence of personality on their competitive abilities, all calves were subjected to personality tests assessing boldness before being exposed to the housing treatment. Our findings indicate that calves assessed as bolder during the pretreatment personality test tended to approach the milk bottle faster. Our results provide additional evidence of the beneficial effects of social housing on dairy calves' behavioral development.

## Highlights

• Pair-housed calves were faster to approach the milk bottle than singly reared calves.
• Pair-housed calves drank longer than singly reared calves in a competitive test.
• Early exposure to a social partner benefits the behavioral development of calves.
• Bolder calves tended to be faster to approach the milk bottle in a competition test.

## Graphical Abstract

**Figure:**
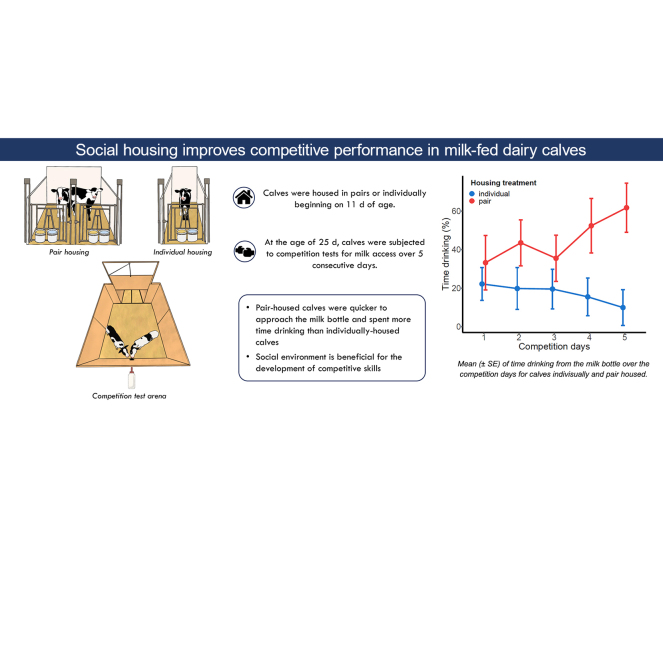
**Summary:** We investigated the effect of early social housing on dairy calves' competitive skills. Calves (3 weeks old) who had been individually or pair housed were subjected to a competition test for milk against a group-reared calf for 5 days. During these tests, pair-housed calves spent, on average, more time drinking from the bottle and were faster to approach the reward than individually housed calves. Two personality tests measuring boldness were also conducted before and after competition tests. Bolder calves tended, on average, to show reduced latencies to the bottle. This work supports early social housing as beneficial for dairy calves.

Within cattle herds, social status influences access to resources such as water (Coimbra et al., 2012) and food (Bica et al., 2019), suggesting a strong link between social skills and welfare (Estevez et al., 2007). Under natural conditions, calves live in complex social groups (Whalin et al., 2021), but on many dairy farms, particularly in North America, preweaning calves are housed individually (USDA, 2016). Previous work suggests that calves are highly motivated for social contact in the first weeks of life (Ede et al., 2022) and numerous studies have shown the welfare and performance costs of depriving calves of an early social environment (reviewed by Costa et al., 2016). Briefly, individually housed calves show increased reluctance to approach an unfamiliar calf (Jensen et al., 1997) and tend to be less dominant when mixed in groups later in life (Warnick et al., 1977; Veissier et al., 1994). Individually housed calves also have reduced behavioral flexibility (Meagher et al., 2015; Whalin et al., 2018), and are more reactive and show increased fearfulness when exposed to novelty (De Paula Vieira et al., 2010, 2012). In addition, weaned calves with prior social experiences are more successful to access grain when forced to compete for food (Duve and Jensen, 2011).

Although social competition has been widely studied in adult dairy cows (Wierenga, 1990), to our knowledge no study has investigated social competition skills of preweaning dairy calves. Furthermore, recent work suggests that personality traits can influence how individual dairy calves cope with stressors (Lecorps et al., 2018), a key factor to be taken into account when investigating a calves' response to social deprivation (Meagher et al., 2015). The aim of our study was to investigate the effect of pair housing beginning at d 11 of life versus individual housing on dairy calves' ability to compete for a highly valued resource (i.e., milk). Calves' individual traits were assessed to control for the influence of personality on their competitive abilities. We predicted that pair-housed calves would be more successful when required to compete for milk access than individually housed calves. We also predicted that bolder calves would be more successful when subjected to a competition test using milk as the food reward compared with calves that are less bold.

This study was conducted from February to August 2022 at the University of British Columbia's Dairy Education and Research Center in Agassiz, British Columbia, Canada. All procedures used were approved by the University of British Columbia Animal Ethics Committee under protocol #A19-0128-A006. Following a power analysis on previous studies investigating the effect of social housing on calf behaviors (*power.t.test* function; De Paula Vieira et al., 2010: latency to approach and time eating; Miller-Cushon and DeVries, 2016: preference for social feeding), we opted to use 9 focal animals per treatment.

Holstein heifers (n = 18) designated as focal animals (birth weight: 37.7 ± 1.0 kg; mean ± SE) were used in this study. All calves were separated from their dam within 12 h of birth, fed 4 L of >50 g/L of IgG colostrum, and then individually housed for the first 10 d of life. On d 5 after birth, all calves were disbudded using caustic paste (see Ede et al., 2020, for full description). At 11 ± 0.32 d old, focal calves were enrolled by block and pseudorandomly allocated within block to a housing treatment for 19.9 ± 0.2 d. Housing treatments consisted of individual (1.2 m × 2.0 m; n = 9 calves) or pair (including a companion calf, 2.4 m × 2.0 m; n = 9 calves). All calves were housed in pens bedded with sawdust. Holstein calves (n = 6; birth weight: 40.5 ± 1.6 kg; 3 heifers and 3 bulls) and Holstein Angus crossbred bulls (n = 3; birth weight: 43.5 ± 0.5 kg) were used as companions (nonfocal) to form the pairs. All focal calves received 8 L/d of whole milk in a nipple bottle divided in 2 meals of 4 L (0800 and 1600 h) and had access to 1 kg/d of starter grain and ad libitum access to water in buckets. Calves were checked at each feeding to ensure access to an adequate amount of milk.

Each focal calf was match paired based on age and birth weight with an unfamiliar “nonfocal” competitor calf (6 Holstein Angus crossbred heifers, 41.3 ± 1.6 kg of birth weight; 4 Holstein Angus crossbred bulls, 39.5 ± 1.8 kg of birth weight; 8 Holstein bulls, birth weight: 40.8 ± 2.1 kg). The competitor calves were group housed at 9.8 ± 0.6 d of age (housed in pens with up to 10 calves) and provided ad libitum access to water, hay, and starter grain via automatic feeders (RIC; Insentec B.V., Marknesse, the Netherlands), and 8 L/d of whole milk through an automatic milk dispenser (CF 1000 CS Combi; DeLaval Inc., Tumba, Sweden). The milk dispenser was fitted with partitions to prevent the drinking calf from being displaced by other calves. To the best of our knowledge, these calves had limited experience with competition for milk access.

Focal calves were subjected to 2 personality tests (**PT1** and **PT2**) at the age of 7.9 ± 0.3 and 30.9 ± 0.3 d to assess their behavioral reactivity to novelty. The personality test arena (12 m × 3.66 m, Figure 1A) had solid walls, was bedded with bark mulch, and was accessed via a start box (1.2 m × 1.8 m). Each focal calf was subjected individually to 3 different sessions, one performed per day for 3 consecutive days, in the following order: (1) open field (empty arena), (2) novel object (PT1: red bin [Uline H-3687R] at arena center; PT2: orange cone [Uline S-19576R] located at arena center), and (3) novel human (unknown human positioned at arena center). At the time of the tests, the calf was gently moved to the start box and then released into the arena for 10 min. Behaviors were recorded for 10 min using a camera (WV-CW504SP, Panasonic, Osaka, Japan) positioned at 7 m above the arena. Vocalizations were recorded live by 2 observers. Distance covered, time spent exploring, latency to approach the object/human and duration of being close (within a radius of 0.9 m), latency to touch the object/human, and duration of contact were collected from the videos by 3 observers. Interobserver reliability was evaluated (R package *irr*, function *icc*) on a random selection of videos assessed by all observers and indicated a good agreement (PT1 and PT2: for each behavior: intraclass correlation coefficient ≥ 0.96, CI = 0.91–1).

**Figure 1:**
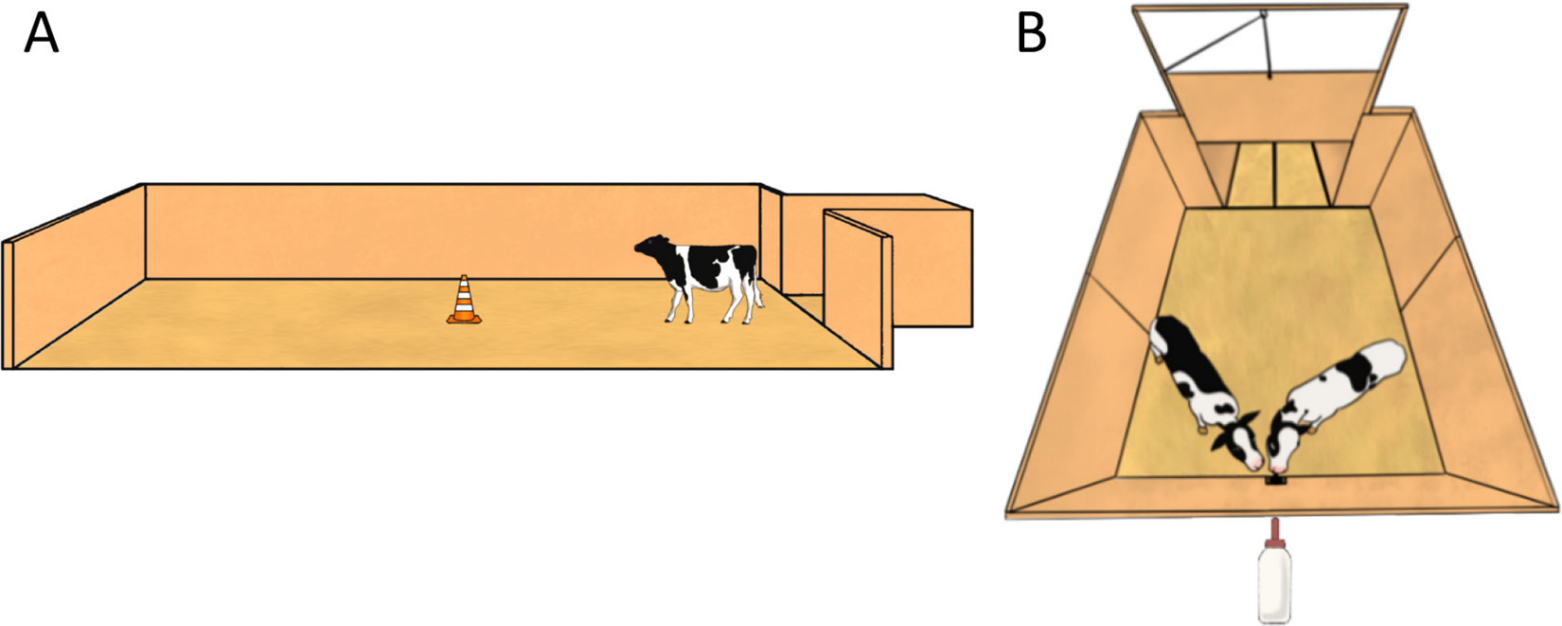
Experimental arenas for (A) personality tests and (B) competition tests. Focal calves (n = 9 individually and n = 9 pair housed) were individually subjected to 2 personality tests to assess their behavioral reactivity to novelty. Each personality test consisted of 3 different sessions: open field, novel object, and novel human. Competition dyads (n = 18; i.e., a focal calf and its assigned competitor) were subjected to a competition test composed of 2 test sessions per day for 5 consecutive days. In the arena, a single bottle filled with 0.5 L of whole milk was accessible.

All calves were individually habituated to the test arena, including having experience drinking from the bottle (Figure 1B), before the competition trials. Training sessions occurred in the morning and started at 12.1 ± 0.1 d after the calves were moved to the housing treatment. Calves were milk-restricted before the training sessions (focal calves were not provided their morning milk ration and competitor calves had no access to milk from the automatic feeders as of 2200 h the night before the training day). The training session began with each calf gently moved to the start box (1.5 m × 1.8 m) with the door to the test arena closed. The test area was 3.7 m × 2.4 m with a single milk bottle, containing 0.5 L of milk, positioned 0.75 m from the floor at the midpoint along the opposing wall from the start box. Calves were allowed to enter the test arena after 2 min of being held in the start box. The training session ended when the calf had consumed all the milk from the bottle; this was repeated 4 times per day. One hour after returning to their home pen, the focal calves were given the remaining 2 L of their morning milk ration and the competitors were allowed access to their daily ration through the automatic feeder. Calves took on average 2.5 ± 0.4 d to reach the learning criterion (start to drink from the bottle within 20 s for 3 consecutive sessions). Calves' latencies to drink were recorded using a camera (GoPro Hero4, GoPro Inc., USA) positioned 1 m above the milk bottle.

Competition tests started the day after the focal calf and its competitor reached the learning criterion (14.0 ± 0.2 d of housing treatment). For the competition test, the focal calf and its competitor were held in the start box for 2 min, before opening the door to the test area where the single bottle filled with 0.5 L of whole milk was located. The test session ended when the milk bottle was empty and the calves were gently moved back into the start box (test session duration: 74.42 ± 2.29 s, range: 36.4–257.7 s). Two test sessions were done per day for 5 consecutive days. Two competition dyads (i.e., the focal calf and its competitor) had a 1-d break between 2 competition tests following diagnosis with scours of one of the calves; the calves were provided electrolytes and in all cases the scours resolved and the testing resumed the following day. Calves were milk-restricted before testing as per the procedure outlined in the training period. Test sessions were recorded as described above. Drinking duration, time spent in proximity of the bottle (i.e., calf nose positioned at less than one-head distance from the milk bottle), and latencies to approach the bottle and to initiate suckling from the teat by focal and competitor calves were collected from videos by 2 observers blind to treatment assignment. Intraobserver reliability was calculated and observers showed a strong agreement for each variable (intraclass correlation coefficient ≥ 0.95, CI = 0.91–1). All statistical analyses were done with *R*, version 4.0.3.

Values of the variables collected in each session (open field, novel object, and novel human) of the personality tests (PT1 and PT2) were averaged to give only one value per variable and per test (Lecorps et al., 2018) for each calf. A principal component analysis (**PCA**) was done to summarize the correlated variables into principal components (*R* package *FactoMineR*; *R* package *factoextra*) using PT1 data. The function *predict.PCA* (*R* package *FactoMineR*) was used to predict the projection of PT2 data of each individual in the PCA setup established with PT1 data. A Pearson correlation test for paired data (*cor.test* function) was performed on scores of both personality tests (PT1 and PT2) to test consistency over time.

Mean comparison tests were realized on calves' latency to drink during the 4 sessions of the last training day using a Fisher-Pitman permutation test for independent data (package *coin*).

Linear mixed-effects models (**LMM**) were performed (*R* package *nlme*; *R* package *lmerTest*) on all behaviors recorded during competition test including session (i.e., first or second daily session) and number of competition days performed as fixed factors, and calf as random factor.

Since we aimed to determine if pair and individually housed calves differed in their access to the milk bottle, the following analysis only included focal calves' data (competitors' data available in Supplementary Materials, https://doi.org/10.5683/SP3/VTGH18). Linear mixed-effects models were performed on (1) the average drinking duration (% compared with the total test duration, i.e., from the gate opening until the bottle was empty of milk) during the 2 daily sessions and (2) their latency to approach the milk bottle (s) as response variables; calf as a random factor; and housing treatment (pair or individual), number of competition days performed, and boldness score as fixed factors. The PT1 boldness score was used to exclude any effect of the housing treatment on calf personality. The *P*-values for LMM were calculated by Monte Carlo sampling with 1,000 permutations, using the *PermTest* function of the *R* package *pgirmess*. For covariate effects of LMM, we calculated marginal pseudo-R^2^ using the function *r.squaredGLMM* (*R* package *MuMIn*). As significant interactions were found between housing treatment and competition day, similar models were conducted on housing treatment separately. Significance and tendency were declared at *P* < 0.05 and *P* < 0.1, respectively.

Our analyses revealed a first principal component with an eigenvalue ≥1 and explaining 51.4% of the variation. This principal component (labeled as boldness) was driven by positive loadings for the distance covered (r = 0.63), the time spent in contact (r = 0.71) and the time spent in proximity with the human and the novel object (r = 0.84), and negative loadings for the latency to approach (r = 0.45) and touch (r = 0.56) the human or object and the time spent exploring (r = 0.41). Calves tended to have consistent boldness scores across time (R^2^ = 0.44, *P* = 0.07).

Calves were trained 4 sessions per day to allow them to habituate to the arena and to learn the placement of the milk bottle; the majority of calves (n = 30) reached the learning criteria in 2 d with 3 of the remaining calves (2 individually housed calves and 1 competitor calf) only requiring one additional day of training. Their counterparts (n = 3) were also subjected to an additional training day. On the last training day, there were no differences in the latency to drink between focal calves (9.4 ± 0.7 s) or competitors (8.6 ± 0.8 s; *P* = 0.47); there were also no differences between pair (9.2 ± 1.1 s) or individually housed calves (9.6 ± 0.9 s; *P* = 0.79).

As daily session order did not affect time drinking (*P* = 0.16) and latency to approach (*P* = 0.21), we used the daily averages in all subsequent analyses. There was an interaction between competition day and housing treatment in time drinking from the bottle (*P* = 0.002; Figure 2A), with the pair-housed calves increasing their time spent drinking over the competition days [marginal R^2^ = 0.06, *P* < 0.01, slope (a) = 6.6], whereas this measure decreased for the individually housed calves (marginal R^2^ = 0.02, *P* = 0.02, a = −2.9).

**Figure 2:**
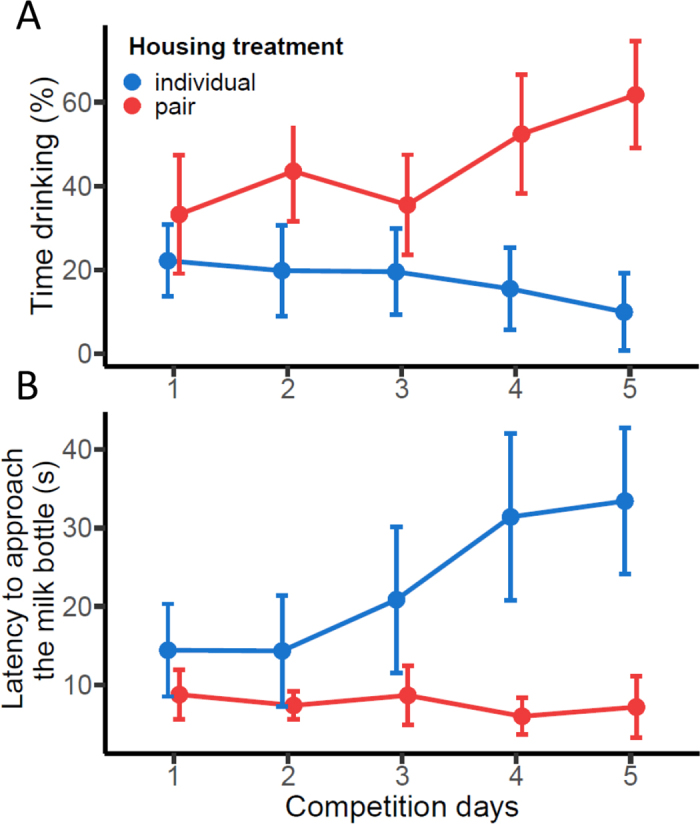
Mean (± SE) of (A) time drinking from the milk bottle (% of total test time) and (B) latencies to approach the milk bottle (s) performed over the competition days for individually (n = 9) and pair-housed calves (n = 9). Each competition dyad (n = 18; i.e., a focal calf [individually or pair housed] and its assigned competitor [group housed]) was subjected to 2 competition sessions per day for 5 consecutive days. Linear mixed-effects models were performed to compare calves' performance over all the competition days.

Latency to approach the bottle was influenced by an interaction between competition day and housing treatment (*P* = 0.01, Figure 2B). Pair-housed calves exhibited consistent latency over time (marginal R^2^ = 0.005, *P* = 0.66, a = −0.5), whereas individually housed calves' latency to approach the bottle increased over the competition days (marginal R^2^ = 0.23, *P* < 0.01, a = 5.5).

Boldness tended to affect the latency to approach the bottle (*P* = 0.06), but we found no effect of boldness on the time drinking from the milk bottle (*P* = 0.90).

During the training sessions, we noted no differences in latency to drink from the bottle, indicating that all calves had similar abilities to approach and drink from the milk bottle. In a social competition setting, pair-housed calves outperformed the individually housed calves: throughout the competition days, individually housed calves decreased their time spent drinking and increased their latency to approach the milk bottle in contrast to pair-housed calves which increased their time drinking and exhibited stable latencies to reach the milk bottle.

The ability to access the milk bottle in a competitive context was affected by the housing treatment. Previous studies investigating individual and social housing during the preweaning period reported that lack of social contact in the individual treatment was associated with lower social rank later on in life when tested at 19 wk (Veissier et al., 1994) or 8 mo (Broom and Leaver, 1978) of age. These results suggest that exposure to a social environment before weaning is influential in the development of competitive skills. Duve and Jensen's (2011) also reported pair-housed calves showing improved performance compared with individually raised calves when subjected to a food competition test. Although our results are overall consistent with their findings, we only identified a difference between treatments beginning on d 4 of testing. These differences are likely explained by Duve and Jensen (2011) employing a group competition test involving 7 calves housed together for 48 h and then tested in the same pen. In contrast, our study involved dyadic contests between calves unfamiliar with each other when exposed to the first competition test and then meeting repeatedly for the 4 subsequent competition tests. Another potential explanation for the differences between the 2 studies is the number of calves involved in the competition test. Having a single opponent versus 6 opponents may have facilitated access to the resource when occupied by the other calf, given that they did not have to outcompete the other calves also trying to gain access, resulting in less difference in time spent drinking between the 2 calves. The similar time spent drinking between individual and pair-housed calves on the first day of competition may also be due to the calves' lack of knowledge about their competitors' skills, which changed as they gained information on the other calf over the subsequent test days (Bradbury and Vehrencamp, 1998).

In the current study, individually housed calves increased their latency to approach the milk bottle over the 5 test days and decreased their time spent drinking, suggesting repeated competitions may have caused these calves to experience a loser effect (i.e., an individual that loses a contest is more likely to lose later contests; Dugatkin, 1997). The loser effect has been argued to influence an individuals' assessment of fighting abilities and fighting costs (Hsu and Wolf, 1999). Individually housed calves' performance suggests a decreased motivation to compete for the resource. This loser effect may thus have affected calves' perception of resource value, as they traded off the costs and benefits of entering the contest (Hsu et al., 2006). The increase in latency to approach the milk bottle by the individually housed calves may also be linked to sensitivity to reward loss. Increasing latencies have been reported when animals were experiencing a reward decrease, with exacerbated phenomenon for individuals housed in a barren environment compared with animals reared in complex environments (rats: Burman et al., 2008; pigs: Luo et al., 2020).

Pair-housed calves showed lower latencies to access the milk reward, compared with individually housed calves, which persisted over the course of the competition test days. This result is consistent with previous work reporting that socially housed calves are less fearful and engage in more social behaviors compared with individually housed calves when mixed with an unfamiliar calf (Jensen et al., 1997, 1999). Enriched environments as social housing have been reported to promote positive affective states (Bučková et al., 2019), which is speculated to increase resilience to environmental changes (Neave et al., 2021). This may explain the improved performance of pair-housed calves when subjected to challenging situations compared with the individually housed calves.

Calves showed a weak consistency in their boldness score across time. Compared with previous studies (Foris et al., 2018; Lecorps et al., 2018), calves were subjected to personality tests at younger ages and were provided a larger surface area to explore during the test, which may have introduced more variation in our results. The PT1 score tended to affect the latency to approach the bottle, with bold calves tending to approach the milk reward more quickly. As competing against an unfamiliar calf for milk access constitutes a novel context, this finding is consistent with the boldness scores obtained using latencies to approach a novel object or human during personality tests. Contrary to our predictions, boldness did not affect time spent drinking, irrespective of the housing treatment, providing evidence that the type of housing provided to calves is more important than individual personality traits when calves are subjected to a competitive situation.

In conclusion, socially housed calves showed reduced latencies to approach the milk bottle and spent more time drinking from the bottle than individually housed calves during a competition test. Our findings provide additional evidence of the detrimental effects of individual housing on the behavioral development of dairy calves.
